# Navigated retrodiaphragmatic/retroperitoneal approach for the treatment of symptomatic kyphoscoliosis: an operative video

**DOI:** 10.3171/2022.3.FOCVID2215

**Published:** 2022-07-01

**Authors:** Michael J. Strong, Joseph R. Linzey, Mark M. Zaki, Rushikesh S. Joshi, Ayobami Ward, Timothy J. Yee, Siri Sahib S. Khalsa, Yamaan S. Saadeh, Paul Park

**Affiliations:** Department of Neurosurgery, University of Michigan, Ann Arbor, Michigan

**Keywords:** lateral access, retrodiaphragmatic approach, operative video, spinal navigation

## Abstract

Retropleural, retrodiaphragmatic, and retroperitoneal approaches are utilized to access difficult thoracolumbar junction (T10–L2) pathology. The authors present a 58-year-old man with chronic low-back pain who failed years of conservative therapy. Preoperative radiographs demonstrated significant levoconvex scoliosis with coronal and sagittal imbalance. He underwent a retrodiaphragmatic/retroperitoneal approach for T12–L1, L1–2, L2–3, and L3–4 interbody release and fusion in conjunction with second-stage facet osteotomies, L4–5 TLIF, and T10–iliac posterior instrumented fusion. This video focuses on the retrodiaphragmatic approach assisted by 3D navigation.

The video can be found here: https://stream.cadmore.media/r10.3171/2022.3.FOCVID2215

## Transcript

Here we present a navigated mini-open retrodiaphragmatic/retroperitoneal approach in a patient with symptomatic degenerative scoliosis.^1–5^

### 0:29 Clinical Presentation.

The patient is a 58-year-old male with over 20 years of low-back pain presenting with progressive debilitating low-back pain and difficulty ambulating that was refractory to conservative management. He denied any radicular leg pain, numbness, or weakness. He also denied any bowel or bladder incontinence or saddle anesthesia. On exam, he was full strength in lower extremities. His reflexes were globally diminished. There was no Hoffman’s, Babinski, or ankle clonus.

### 0:54 Neuroimaging Findings.

Standing full-length x-rays demonstrate a significant levoconvex scoliosis with an L1 to L5 Cobb angle of 46°. He has both coronal and sagittal imbalance with a 10-cm rightward shift of his C7 plumb line from the central sacral vertical line as well as positive 12-cm sagittal imbalance. Comparing the standing x-rays to the supine CT scout images reveals a stiff deformity. CT lumbar spine demonstrates collapsed disc space with multiple bridging osteophyte complexes. Lumbar MRI reveals multilevel stenosis, most significant at L4–5, where there is severe central canal stenosis, and L3–4, where there is also severe central canal stenosis along with multilevel foraminal and lateral recess stenoses.

### 1:39 Operative Plan.

Our operative plan was for a staged approach, with the first stage involving a left-sided retrodiaphragmatic and retroperitoneal approach for T12–L4 lateral interbody fusions. This was followed with a second-stage T10–ilium instrumentation and fusion, facet osteotomies, and left L4–5 TLIF. The focus of this video will be on the retrodiaphragmatic approach to the T12–L1 disc space, as well as highlighting the utility of navigation in performing a lateral osteotomy across a fused portion of the disc space at L1–2.

### 2:12 Positioning.

After general anesthesia, the patient is positioned in lateral decubitus position with the convex side up on a Jackson table. A large gel roll is placed under the torso and the patient is secured into place with tape. This maneuver elevates the rib cage cranially as well as opens up the disc spaces. Approaching on the concave side poses more surgical challenge since the disc spaces are collapsed and there are numerous bridging osteophytes. The hips and knees are flexed to prevent strain on the psoas muscle.

### 2:39 Planning of Surgical Incision Using Navigation System.

The patient is prepped and draped in usual sterile fashion. A stab incision overlying the superior-most portion of the iliac crest was made and an iliac pin with a stealth reference frame was impacted into the bone. A 3-dimensional image of the spine was obtained. The navigation system is used to trace out the skin incision, which is centered on the thoracolumbar junction and the 11th rib and measured approximately 2.5 inches. This surgical incision was used for the T12–L1 and L1–2 lateral interbody fusions. Another similar sized incision was made for the L2–3 and L3–4 lateral interbody fusions, which will not be depicted in this operative video.

### 3:18 Rib Dissection and Removal.

The skin is incised and electrocautery is used to expose the 11th rib going through the latissimus dorsi and external oblique muscles. The 11th rib is identified and the intercostal muscle is dissected off the rib to allow room for insertion of a rib elevator, which will separate the rib from the underlying endothoracic fascia and diaphragm. When dissecting the muscle off the rib caudally, care must be taken not to disrupt the neurovascular bundle that runs inferior to the rib. Once separated, rib cutters are used to cut a portion of the rib approximately 5 cm to allow room for exposure to the thoracolumbar junction. This piece of bone was saved and used later as local autograft bone.

### 3:56 Demonstration of Diaphragm Dissection Off Rib.

A plane is developed between the intercostal muscle cuff and the diaphragm using blunt dissection. The intercostal muscle cuff with the neurovascular bundle is lifted and the diaphragm is exposed underneath. Blunt dissection is continued in the direction of the 12th rib. Diaphragm was dissected dorsally as well as inferiorly. The T12 rib is identified using the navigation pointer. This confirms that our trajectory is correct. The diaphragm is visualized attaching under the 12th rib; blunt dissection is utilized to free the diaphragm from this rib. Navigation confirms we are under the 12th rib approaching the vertebral body.

### 4:37 Retrodiaphragmatic Approach With Placement of Tubular Retractor.

A handheld retractor is placed on the diaphragm to elevate it cranially and anteriorly. This allows for an adequate corridor to the T12–L1 disc space. Navigation confirms that we are right over the T12–L1 disc space. Blunt dissection is continued to expose the psoas muscle and the underlying disc space. The navigated pointer is anchored into the disc space and confirmed using the navigated system. Sequential dilation through the psoas is utilized and a tubular retractor is docked on the disc space. EMG is utilized with the navigated pointer as it traverses the psoas and anchors into the disc space. Of note, the psoas muscle bulk is variable and may be atretic at the T12–L1 disc space. Any remaining psoas muscle fibers are mobilized and displaced using a lateral retractor.

### 5:26 Performance of Annulotomy, Discectomy, and Placement of Interbody Cage.

A bayonetted knife is used to make an annulotomy into the disc, followed by standard discectomy and endplate preparation. Navigated trials are inserted into the disc space to determine appropriate sizing of the interbody fusion cage. A biologic to enhance fusion is used in conjunction with the allograft bone. The interbody cage packed with allograft bone is impacted into the disc space utilizing spinal navigation. After placement of the cage, visual inspection is performed to confirm adequate placement of the cage and to achieve hemostasis prior to retractor removal.

### 5:59 Lateral Osteotomy Using Navigation.

Since the L1–2 disc space was partially fused, a navigated Cobb was advanced across this fused portion until a complete osteotomy was performed through the lateral osteophyte complex.

### 6:09 Closure of Incision.

Using this approach, the diaphragm does not have to be surgically reapproximated. The surgical incision is closed in layers, making sure to reapproximate both the external oblique and latissimus dorsi muscles. Skin was closed with skin glue.

### 6:20 Key Points.

In this video, we illustrated a case of symptomatic thoracolumbar scoliosis with both sagittal and coronal imbalance treated with a retrodiaphragmatic/retroperitoneal approach for lateral interbody fusions and posterior osteotomies, instrumentation, and fusion. We focused on the retrodiaphragmatic approach to the T12–L1 disc space and navigated osteotomy. Utilizing 3D navigation in this case offered several advantages, including planning our incision, assisting in intraoperative localization as we were dissecting the diaphragm off the rib, completing a lateral osteotomy across a fused portion of the disc space, and guiding placement of an interbody cage.

### 7:00 Postoperative Course.

The patient had an uncomplicated hospital course and was discharged on hospital day 5 to home.

### 7:05 Follow-Up.

At the 6-month follow-up visit, he reported significant improvement in his back pain. Postoperative scoliosis x-rays demonstrate significant improvement in alignment with the L1–5 Cobb angle of 46° preoperatively to 8° postoperatively. His coronal offset was markedly improved from 10 cm to half a centimeter. Also, his sagittal imbalance corrected from 12 cm to 4.5 cm postoperatively.

## Acknowledgments

We would like to thank Tom Cichonski for assistance with the preparation of this video.

## Disclosures

Dr. Park reported personal fees from Globus, NuVasive, DePuy Synthes, and Accelus; and grants from DePuy Synthes, Cerapedics, SI-BONE, and ISSG, outside the submitted work.

## Author Contributions

Primary surgeon: Park. Assistant surgeon: Khalsa, Saadeh. Editing and drafting the video and abstract: Park, Strong, Linzey, Ward, Saadeh. Critically revising the work: all authors. Reviewed submitted version of the work: all authors. Approved the final version of the work on behalf of all authors: Park. Supervision: Park.
